# Optimal timing of enteral nutrition initiation in critically ill patients: a network meta-analysis

**DOI:** 10.3389/fnut.2026.1722626

**Published:** 2026-02-06

**Authors:** Ming Dai, Hong-Wei Yang, You Zhou, Qian Guo, Yuan Meng, Yun Lei Sun, Pei-Ya Hu

**Affiliations:** 1 Department of Intensive Care Unit, The First Affiliated Hospital of Zhejiang Chinese Medical University (Zhejiang Provincial Hospital of Chinese Medicine), Hangzhou, China; 2 Department of Nursing, The First Affiliated Hospital of Jiamusi University, Jiamusi, Heilongjiang, China

**Keywords:** critically ill patients, enteral nutrition initiation, mortality, network meta-analysis, optimal timing

## Abstract

**Background:**

Nutritional support is pivotal in managing critically ill patients. Enteral nutrition, which preserves intestinal mucosal barrier function and modulates immune-metabolic homeostasis, is the preferred nutritional support strategy. However, the optimal timing for initiating EN remains controversial: some studies advocate early initiation (within 24–48 h), while others suggest delayed initiation (beyond 48 h), resulting in inconsistent clinical practices and conflicting guideline recommendations. This highlights the need for high-quality evidence to clarify the optimal EN initiation window.

**Objective:**

This network meta-analysis aims to systematically compare the effects of five EN initiation timings on key clinical outcomes in critically ill patients, rank their efficacy, and identify the optimal initiation window, thereby providing evidence-based guidance for clinical practice.

**Methods:**

We conducted a network meta-analysis following the PRISMA-NMA statement, including randomized controlled trials (RCTs) comparing the five EN initiation timings in critically ill adults. Databases (PubMed, Embase, Web of Science, Cochrane Library) were searched from inception to September 2024. Data analysis was performed using Stata 16.0 and RevMan 5.4, with efficacy ranked via the surface under the cumulative ranking curve (SUCRA).

**Results:**

Fifteen RCTs were included, involving five EN initiation timings: <24 h, 24–48 h, 48–72 h, 72–96 h, and >96 h. No statistically significant differences in mortality or ICU length of stay were observed between any two timings. However, SUCRA ranking showed that 24–48 h initiation had the highest probability of reducing mortality (SUCRA = 67.0), while >96 h initiation was most likely to shorten ICU length of stay (SUCRA = 75.2).

**Conclusion:**

Initiating EN beyond 96 h was associated with the shortest ICU stay, while initiating it within 24–48 h may be associated with lower mortality. Based on the current limited evidence, the 24–48 h window may be relatively optimal for improving clinical outcomes in critically ill patients. However, given the small number of included studies and low to moderate evidence quality, this conclusion requires validation in large, high-quality RCTs.

**Systematic review registration:**

https://www.crd.york.ac.uk/PROSPERO/view/CRD42024581390. identifier PROSPERO (CRD42024581390).

## 1 Introduction

Critically ill patients are characterized by unstable or potentially unstable vital signs, with one or more organ systems impaired, thereby posing an immediate life threat (1). Advances in critical care have significantly improved ICU treatment standards, contributing to a steady decline in mortality over recent decades (2). However, these patients frequently develop severe malnutrition due to their inherent hypercatabolic and hypermetabolic states, combined with inadequate nutrient intake during critical illness (3). In ICUs, the prevalence of malnutrition ranges from 38 to 78%, which independently exacerbates poor outcomes (4). Specifically, malnutrition in critically ill patients is associated with prolonged mechanical ventilation, impaired wound healing, increased complications, extended hospital stays, and elevated mortality (5, 6). Long-term consequences include reduced quality of life and substantial economic burdens on families (5, 7). Therefore, effective clinical nutritional support is critical for improving malnutrition and clinical outcomes in critically ill patients (8).

Nutritional support modalities include enteral nutrition (EN) and parenteral nutrition (PN). EN delivers nutrients via the gastrointestinal tract, whereas PN provides nutrients intravenously, bypassing the gut (9). EN preserves the structure and function of the gastrointestinal tract by simulating physiological feeding, promoting intestinal motility, supporting digestive enzyme secretion, and reducing bacterial translocation (10). Compared to PN, EN is associated with fewer severe complications (e.g., catheter-related infections, metabolic disturbances, and hepatic dysfunction), though minor adverse events such as diarrhea or bloating may occur (11). Therefore, when the gastrointestinal tract is functioning properly, EN is the preferred method of nutritional support.

A landmark meta-analysis found that EN initiated within 24 h of ICU admission significantly improved outcomes and reduced mortality (12). In contrast, a recent meta-analysis reported that EN started within 24–48 h did not significantly shorten ICU or hospital stays in critically ill patients (13), highlighting conflicting evidence. Guidelines also differ: the 2016 SCCM/ASPEN guidelines emphasize that EN better preserves intestinal mucosal integrity, stabilizes gut microbiota, and regulates immune-metabolic function compared to PN, advocating EN as the preferred route (14–17). They recommend initiating EN within 24–48 h of ICU admission once hemodynamic stability is achieved (18). Conversely, the European Society for Clinical Nutrition and Metabolism (ESPEN) guidelines suggest considering nutritional support for ICU patients hospitalized beyond 48 h (3), reflecting inconsistent recommendations.

Network meta-analysis extends traditional pairwise meta-analysis by enabling simultaneous comparison of multiple interventions and ranking their effectiveness and safety based on integrated direct and indirect evidence (19). This approach enhances the accuracy of results by leveraging a broader evidence network, making it well-suited to resolve controversies involving multiple time windows. Accordingly, we conducted a network meta-analysis to systematically compare the effects of five EN initiation timings on clinical outcomes in critically ill patients, aiming to identify the optimal window and provide evidence-based guidance for clinical practice.

## 2 Methods

Our study adhered to the Preferred Reporting Items for Systematic Reviews and Meta-Analyses (PRISMA-NMA) statement for network meta-analyses (20). The study protocol was registered on the PROSPERO platform (registration number: CRD42024581390; Registration Date: September 4, 2024). The full registration protocol and study details are publicly accessible at: https://www.crd.york.ac.uk/PROSPERO/view/CRD42024581390.

### 2.1 Inclusion and exclusion criteria

The inclusion and exclusion criteria were developed according to the PICOS principle. Included studies were: (1) Population: Adult patients (aged ≥ 18 years) admitted to the ICU who required EN. We included studies enrolling a broad spectrum of critically ill patients, such as those with trauma (e.g., blunt trauma, traumatic brain injury), post-operative status (e.g., abdominal or thoracic surgery), severe infections (e.g., septic shock), neurological injuries, severe acute pancreatitis, and burns. (2) Intervention: The interventions in this study included different timing points for initiating enteral nutrition in critically ill patients, specifically categorized as <24 h, 24–48 h, 48–72 h, 72–96 h, and >96 h. (3) Outcomes: mortality rate; ICU length of stay. (4) Study design: randomized controlled trials (RCTs). Excluded studies were: (1) Those failing to clearly delineate the timing of enteral feeding initiation; (2) Those involving patients receiving parenteral nutrition or enteral feeding combined with parenteral nutrition; (3) Studies written in a language other than English; (4) Studies with incomplete data or source data or full-text files could not be obtained after contacting the corresponding author; (5) Conference abstracts duplicated across multiple events or published excessively. Detailed baseline characteristics of the included patients, including the distribution of clinical conditions, are summarized in Table 1.

**Table 1:** Characteristics of included studies.

Study	Country	Sample size③ (C/T)		Age③ (C/T)		Gender④ (M/F)③ (C/T)		Disease type	Enteral feeding timing③ (C/T)		Outcome
(35)	USA	19	19	41 ± 18	44 ± 22	8/11	14/5	Blunt trauma	72–96 h	<24 h	① ②
(36)	Canada	15	13	61 ± 12	64 ± 11	11/4	11/2	Abdominal or thoracic surgery	>96 h	<24 h	②
(37)	Slovenia	14	14	44.7 ± 15.9	38.4 ± 15.6	10/4	14/0	Multiple organ failure (MOF) in multiply injured patients.	24–48 h	<24 h	②
(38)	USA	15	12	36 ± 11	30 ± 13	10/5	9/3	Severe closed-head injuries	>96 h	48–72 h	①②
(39)	USA	75	75	59.1 ± 19.0	56.5 ± 15.6	35/40	28/47	Mechanically ventilated patients	>96 h	<24 h	①②
(40)	Germany	40	40	67 ± 13	62 ± 18	17/23	16/24	After percutaneous endoscopic gastrostomy	24–48 h	<24 h	①
(41)	Slovenia	25	27	41.5 ± 16.8	42.6 ± 17.9	21/4	24/3	Gastric intolerance and subsequent pneumonia	24–48 h	<24 h	②
(42)	USA	13	14	49 ± 19	44 ± 24	10/3	9/5	Burn injury	>96 h	<24 h	①②
(27)	Australia	14	14	56.3 ± 3.4	54.9 ± 3.3	10/4	8/6	Critically ill patients	>96 h	<24 h	①②
(43)	Thailand	29	27	37.8 ± 53.0	36.6 ± 49.3	2/27	8/19	Cerebral malaria	48–72 h	<24 h	①②
(44)	Greece	25	34	33.30 ± 12.96	36.13 ± 14.72	21/4	26/8	Traumatic brain injury	48–72 h	24–48 h	①②
(45)	China	30	30	42.8 ± 12.8	43.9 ± 13.2	18/12	20/10	Severe acute pancreatitis	>96 h	24–48 h	①②
(46)	USA	16	15	56 ± 16	64 ± 14	8/8	10/5	Septic shock	48–72 h	<24 h	①
(47)	China	43	44	⑤	⑤	⑤	⑤	Critically ill patients	24–48 h	<24 h	②
(48)	China	75	77	45.48 ± 9.32	46.37 ± 9.94	40/35	44/33	Traumatic intracerebral hemorrhage (TICH)	48–72 h	24–48 h	①

① Mortality; ② ICU Length of Stay; ③ (C/T)C: Control Group, T: Treatment Group; ④ (M/F)M: Male, F: Female; ⑤: Not mentioned.

### 2.2 Search strategy

We conducted a network meta-analysis by searching four databases: PubMed, Embase, Web of Science, and Cochrane Library databases were searched from inception to September 2024. Only English-language articles published during this timeframe were included. Reference lists from all selected primary studies and review articles were also examined for additional relevant citations. A detailed search strategy is outlined in Appendix 1. The main search strategies were as follows: (“Intensive Care Units”[MeSH] OR”critical care unit”[Text Word]) AND (“Enteral Nutrition”[MeSH] OR “Enteral Feeding”[Text Word]) AND (“Randomized controlled trial”[Text Word]).

### 2.3 Study selection

After removing duplicate records, two researchers (MD and YM) separately screened the titles and abstracts of the related articles in the initial stage. Then, the full-text articles were assessed to check if they met all the inclusion and exclusion criteria. Any disagreement or uncertainty between the two researchers was solved by discussion or consultation with a third researcher (PYH).

### 2.4 Data extraction

Two researchers (MD and HWY) separately extracted data using a Excel 2021 form, which was designed based on the guidelines from the Cochrane Handbook for Systematic Reviews of Interventions (21). Any disagreements were resolved through rechecking the original research and discussion with another researcher (PYH). For each study, we extracted the following information: author(s), age, gender, year of publication, title, study design, sample size, timing of initiating enteral nutrition, and corresponding outcome variables.

### 2.5 Risk of bias and grade of evidence assessment

Two researchers (MD and YM) used the revised Cochrane Risk of Bias tool (version 2), and any disagreements were resolved through discussion with another reviewer (PYH) (22). Bias was assessed across five domains. The risk of bias for each domain was assessed as high risk, low risk, or some concern. We used the Grading of Recommendation, Assessment, Development, and Evaluation criteria to evaluate the overall evidence quality in five aspects: (1) bias risk, (2) inconsistency, (3) indirectness, (4) imprecision, and (5) publication bias. The evidence quality can be divided into high, moderate, low, or very low levels (23).

### 2.6 Statistical analysis

First, pairwise meta-analyses were performed using RevMan 5.4 to conduct direct comparisons. In data analysis, binary variables were expressed using RR (risk ratio) values, and continuous variables were expressed using MD (mean difference) values (21). To assess between-study heterogeneity, researchers used the *I^2^* statistic and visually inspected forest plots. When *I^2^* = 0, it indicated that no heterogeneity was present. Low, medium, and high degrees of heterogeneity were defined by *I*^2^ values of 25, 50, and 75%, respectively (24). When *p* > 0.1 and *I^2^* < 50%, heterogeneity was considered low, and the fixed effects model was used for analysis. If *I^2^* > 50%, significant heterogeneity was present, and the random effects model was applied for the combined analysis.

Second, network meta-analysis was performed using STATA 16.0, with data analyzed under the frequentist framework (25). In the network plot, each node represents an intervention, and the line connecting any two nodes indicates direct comparisons between these interventions. The width of each line corresponds to the number of studies comparing the two interventions. Multiple closed loops are formed among interventions. Overall inconsistency was evaluated using the inconsistency factor (IF) and its 95% confidence interval (95%CI), while local inconsistency was detected using the node-splitting method. Local inconsistency was considered to exist when *p* < 0.05. Various intervention types were ranked based on cumulative probabilities derived from the surface under the cumulative ranking curve (SUCRA). SUCRA values range from 0 to 1, with higher values indicating a greater likelihood of being the most effective intervention (26).

## 3 Results

### 3.1 Search results

**Figure 1:**
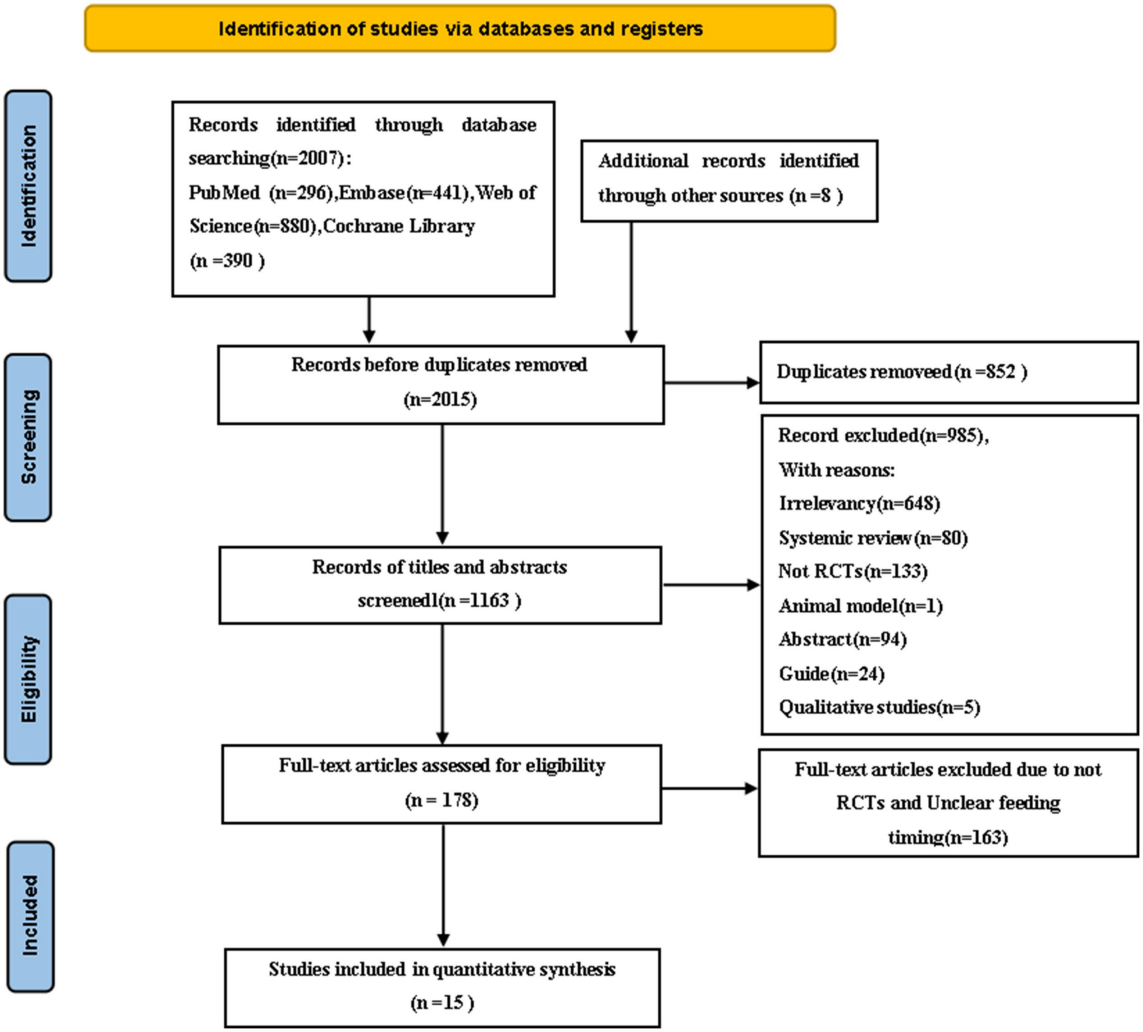
Flowchart of all studies identified, included, and excluded following the PRISMA 2020 statement.

The literature screening process and results are shown in Figure 1. Initially, we identified a total of 2015 studies. After eliminating duplicates in articles, titles, and abstracts, we selected 178 full-text articles for detailed review. Finally, 15 studies were included in the network meta-analysis.

### 3.2 Characteristics of the included studies

The characteristics of the included RCTs were summarized in Table 1. A total of 15 RCTs, involving 903 patients from nine countries, were included, and five different time points of enteral feeding in critically ill patients were considered.

### 3.3 Risk of bias and grade of evidence

**Figure 2:**
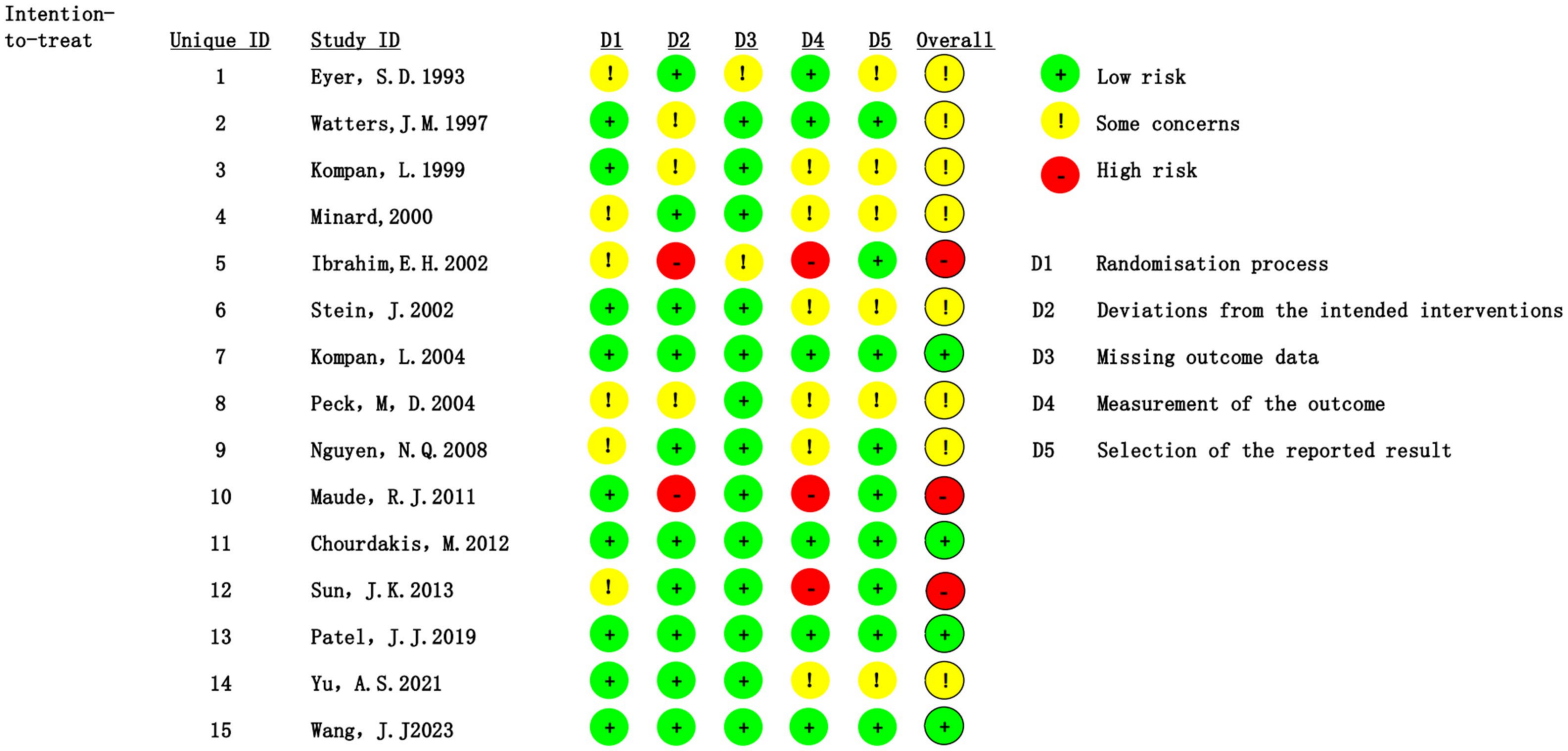
Assessment of the risk of bias summary.

Using the ROB 2.0 tool, we assessed the risk of bias as follows: 20% of studies were rated as high risk, 53.3% had some concerns, and 26.7% were considered low risk. The randomization process was generally well conducted, with 60% of studies rated as low risk. However, concerns remained for 40% of studies, primarily due to insufficient details on allocation concealment. Issues related to deviations from the intended interventions were noted, with 13.3% of studies rated as high risk—mainly because blinding of researchers or participants was not implemented, potentially leading to performance bias. Regarding outcome measurement bias, 20% of studies were rated as high risk, largely because it was unclear whether outcome assessors were aware of the interventions received by the participants, as illustrated in Figure 2. The results of the Grading of Recommendations, Assessment, Development, and Evaluation (GRADE) are presented in Appendix Table 2.

### 3.4 Traditional pairwise meta-analysis

**Figure 3:**
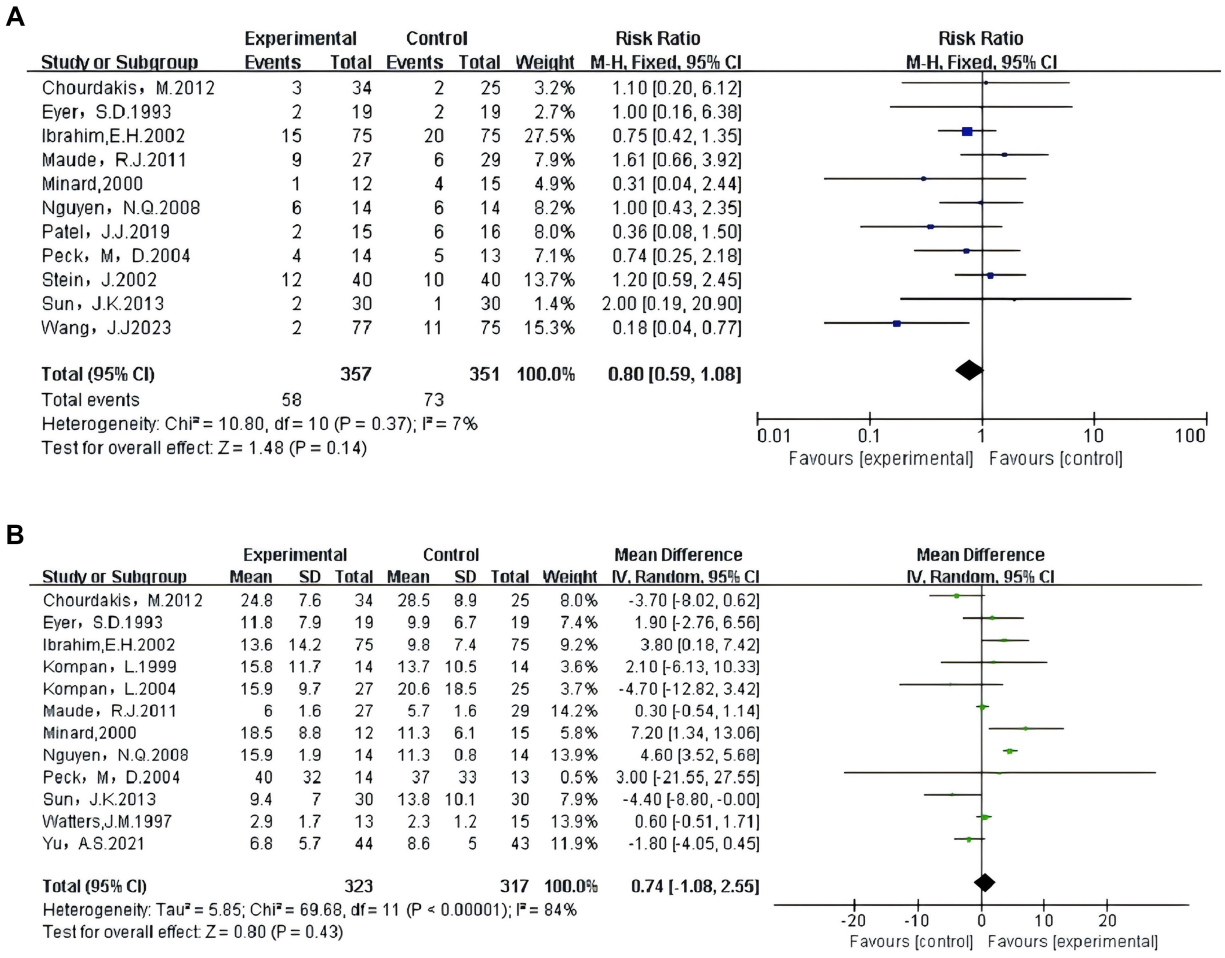
**(A)** Traditional pairwise meta-analysis of mortality rate. **(B)** Traditional pairwise meta-analysis of length of ICU stay.

The results of the traditional pairwise meta-analysis are shown in Figures 3A,B.

A total of 15 studies were included in this systematic review, 11 of which assessed the impact of different enteral feeding timings on mortality in critically ill patients. The meta-analysis of these 11 studies, comprising 357 participants in the treatment group and 351 in the control group, revealed a risk ratio of 0.80 (95% CI: 0.59, 1.08; *I*^2^ = 7%; *p* = 0.14). Given the low heterogeneity observed in this outcome, all eligible studies were then included in the network meta-analysis for further comparative analyses. Additionally, 12 studies reported the effects of different enteral feeding timings on ICU length of stay for critically ill patients. The meta-analysis of these studies, which included 323 participants in the treatment group and 317 in the control group, showed a mean difference of 0.74 (95%CI: −1.08, 2.55; *I*^2^ = 84%; *p =* 0.43).

Sensitivity analysis revealed that after excluding Study 9 (27), the heterogeneity decreased to *I^2^* = 55%. Quality assessment using the ROB 2.0 tool indicated that this study was at high risk of bias, suggesting it might be the primary source of heterogeneity. Consequently, we excluded this study and incorporated the remaining qualified studies into the final network meta-analysis.

### 3.5 Network meta-analysis

#### 3.5.1 Network plots

**Figure 4:**
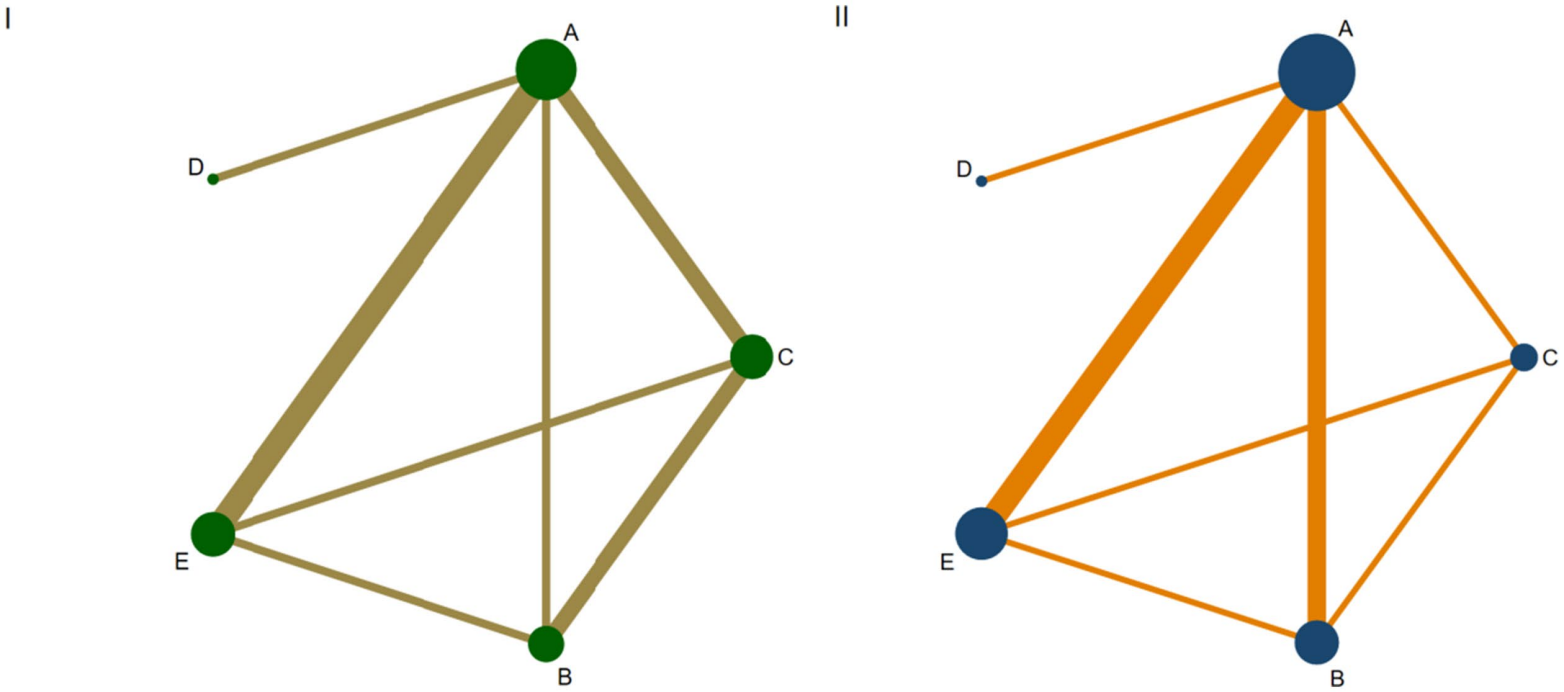
**(I,II)** Network plot of outcome indicators. **(I)** Mortality rate; **(II)** Length of ICU stay; A: <24 h; B: 24–48 h; C: 48–72 h; D: 72–96 h; E: >96 h.

The network plot illustrates the network structure and the number of studies on interrelationship among different enteral feeding timing. The most common enteral feeding timing was A, while the least frequent was D. A total of 15 studies reported on various timings of enteral feeding in critically ill patients, involving five intervention times, which collectively formed eight triangular closed loops, as shown in Figures 4I,II.

#### 3.5.2 Basic hypothesis testing

Loop inconsistency tests, inconsistency models, and node splitting methods were employed to examine the inconsistency of each outcome indicator. A consistency inspection showed that most *p* > 0.05, indicating good agreement and no significant overall inconsistency, as shown in Appendix 2.

#### 3.5.3 Effect on mortality rate

**Figure 5:**
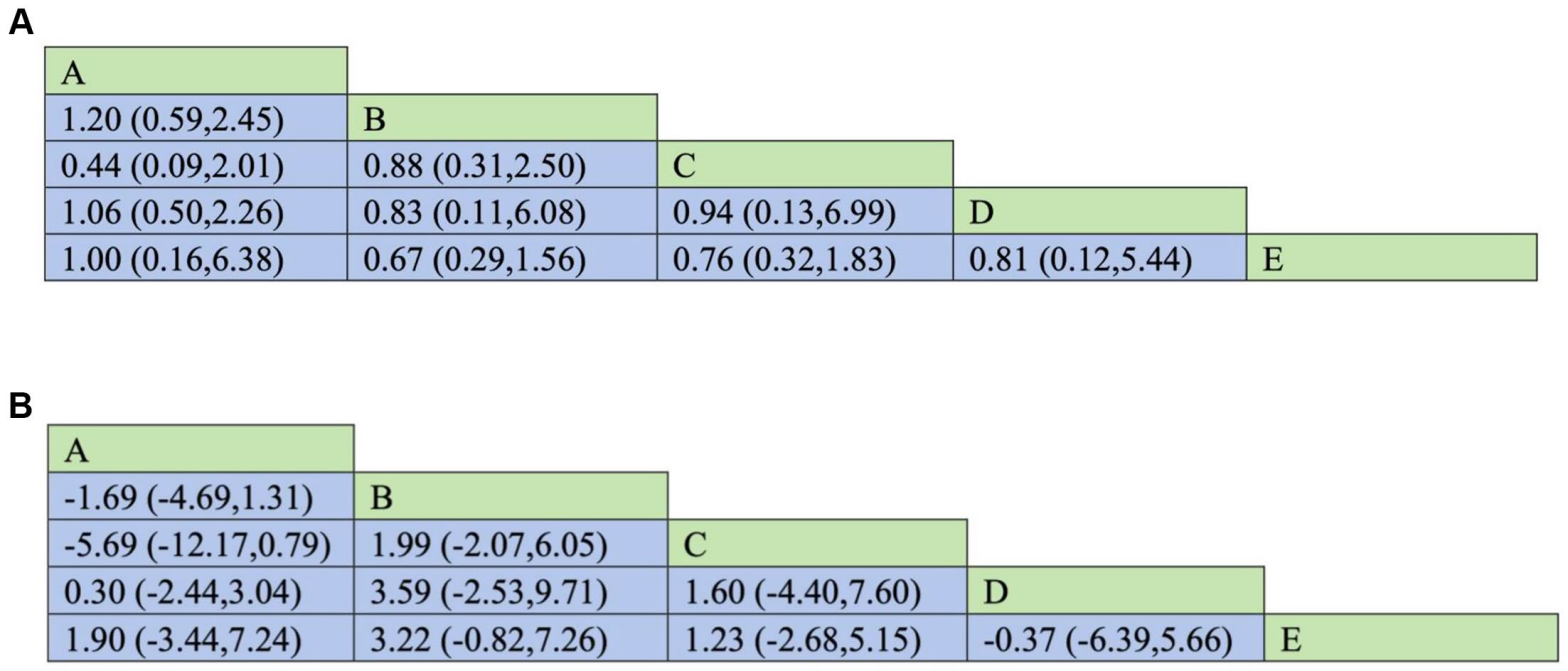
**(A)** Revised. Network meta-analysis results of different intestinal nutrition timing on mortality in critically ill patients [RR (95% CI)]. A: <24 h; B: 24–48 h; C: 48–72 h; D: 72–96 h; E: >96 h. **(B)** Revised. Network meta-analysis results of different enteral nutrition timing on the length of hospital stay for critically ill patients [MD (95% CI)]. A: <24 h; B: 24–48 h; C: 48–72 h; D: 72–96 h; E: >96 h.

Pairwise comparisons evaluating the impact of different enteral feeding timings on the primary outcome (mortality) in critically ill patients are presented in Figure 5. Results indicated no statistically significant differences in mortality between the following enteral feeding timings: <24 h vs. 24–48 h; 24–48 h vs. 48–72 h; 48–72 h vs. 72–96 h; 72–96 h vs. > 96 h; and <24 h vs. > 96 h. The respective results were: (RR = 1.20, 95%CI: 0.59, 2.45), (RR = 0.88, 95%CI: 0.31, 2.50), (RR = 0.94, 95%CI: 0.13, 6.99), (RR = 0.81, 95%CI: 0.12, 5.44), and (RR = 1.00, 95%CI: 0.16, 6.38).

#### 3.5.4 Effect on ICU length of stay

Pairwise comparisons evaluating the impact of different enteral feeding timings on the primary outcome (ICU length of stay) in critically ill patients are presented in Figure 5. Results indicated no statistically significant differences on ICU length of stay between the following enteral feeding timings: <24 h vs. 24–48 h; 24–48 h vs. 48–72 h; 48–72 h vs. 72–96 h; 72–96 h vs. >96 h; and <24 h vs. >96 h. The respective results were: (MD = −1.69, 95%CI: −4.69, 1.31); (MD = 1.99, 95%CI: −2.07, 6.05); (MD = 1.60, 95%CI: −4.04, 7.60); (MD = −0.37, 95%CI: −6.39, 5.66); (MD = 1.90, 95%CI: −3.44, 7.24).

Analysis using the Surface Under the Cumulative Ranking Curve (SUCRA) revealed distinct population-specific trends and ranking probabilities. For mortality outcomes, the SUCRA values for the five EN initiation timings were as follows: <24 h (SUCRA = 51.7%), 24–48 h (SUCRA = 67.0%), 48–72 h (SUCRA = 55.4%), 72–96 h (SUCRA = 49.8%), and >96 h (SUCRA = 26.1%). For ICU length of stay, the SUCRA values were: <24 h (SUCRA = 41.3%), 24–48 h (SUCRA = 12.3%), 48–72 h (SUCRA = 49.4%), 72–96 h (SUCRA = 71.8%), and >96 h (SUCRA = 75.2%).

### 3.6 Publication bias analysis

**Figure 6:**
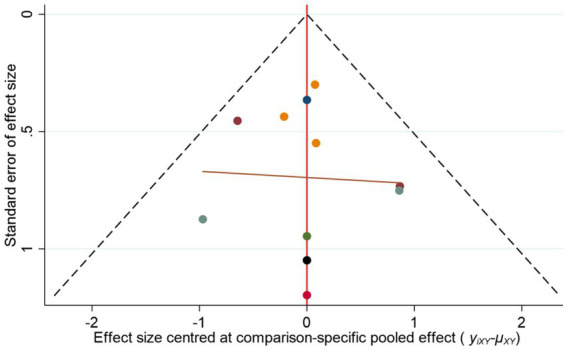
Funnel plot for mortality rate.

A funnel plot was generated to assess publication bias for the mortality outcome. The results showed that study points were approximately symmetrically distributed on both sides of the central axis, suggesting a low likelihood of publication bias, as shown in Figure 6.

**Figure 7:**
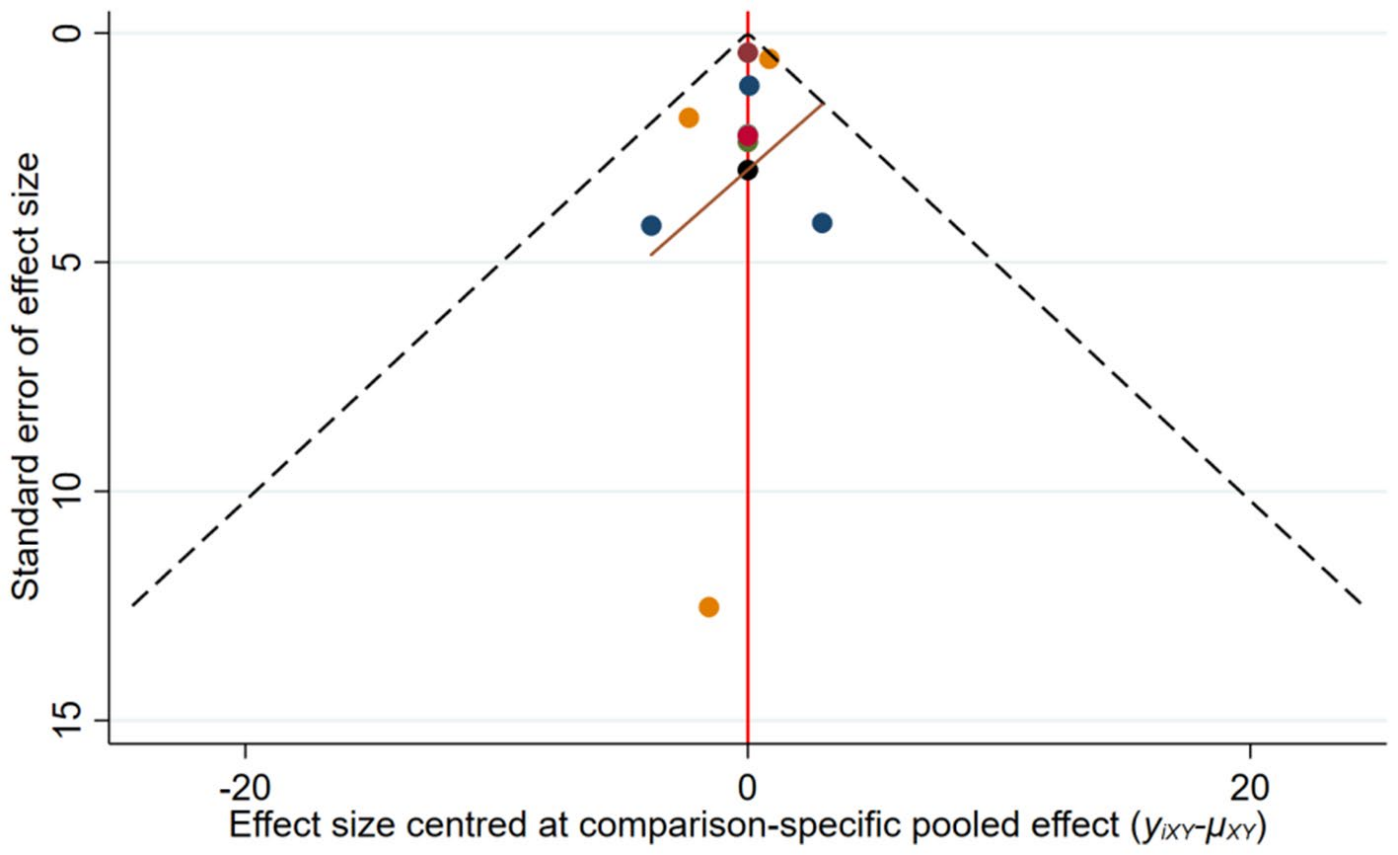
Funnel plot for the length of ICU stay.

A funnel plot was generated to assess publication bias for the ICU length of stay. The results showed that study points were approximately symmetrically distributed on both sides of the central axis, suggesting a low likelihood of publication bias, as shown in Figure 7.

## 4 Discussion

This study found no statistically significant differences in mortality or ICU length of stay across the five EN initiation timings in critically ill patients (all *p* > 0.05). However, SUCRA rankings revealed notable population-specific trends: initiating EN within 24–48 h may reduce mortality in certain patient subgroups, while delaying EN initiation beyond 96 h might shorten ICU length of stay in some populations.

The advantage of initiating EN within 24–48 h stems from the dual mechanisms of “stress regulation and gut protection.” Animal studies have demonstrated that guinea pigs with experimental burns who were fed within 2 h of injury exhibited significantly lower 24 h urinary vanillylmandelic acid excretion levels compared to those with delayed feeding at 72 h. This biomarker difference directly indicates that early feeding can suppress the overactivation of the sympathetic-adrenal medullary system, reduce catecholamine secretion, and thereby alleviate the prolonged hypermetabolic stress state post-trauma, preventing metabolic disturbances and immune dysregulation (28). This mechanism has been indirectly validated in human trauma patients. Clinical studies have shown that patients who initiated EN within 24–48 h after trauma had significantly lower serum catecholamine levels and a 30% reduction in the incidence of immune dysregulation compared to the delayed feeding group (29).

A mechanistic study on SAP (severe acute pancreatitis) rats demonstrated that early EN significantly upregulated the expression of mucosal address in cell adhesion molecule-1 (MAdCAM-1) in the intestinal mucosa and increased the infiltration of CD4^+^ and CD8^+^ T cells. This change directly alleviated pathological intestinal damage in SAP rats, reduced serum endotoxin levels and bacterial translocation, and ultimately correlated with decreased mortality (30). This mechanism is also applicable to human SAP patients: clinical studies have shown that SAP patients who initiated EN within 48 h of onset had significantly lower rates of enterogenous infections and a reduced incidence of multiple organ dysfunction syndrome (MODS) compared to the delayed feeding group (31). This supports the mortality benefit of initiating EN within 24–48 h for SAP patients, which is fully consistent with the SUCRA trend of reduced mortality in the 24–48 h group in this study. The potential advantages of initiating EN within 24–48 h primarily manifest in hemodynamic stability, relatively normal gastrointestinal function, and the capacity to reduce mortality by protecting the intestinal barrier and suppressing stress responses.

The advantage of initiating EN beyond 96 h lies in avoiding aspiration pneumonia, shortening the infection control cycle, and reducing feeding-related complications. For cerebral malaria patients in resource-limited settings who cannot undergo endotracheal intubation and remain comatose within the first 24 h of admission, the core risk of initiating EN is “aspiration leading to aspiration pneumonia.” From a pathophysiological perspective, in a state of deep coma, the swallowing reflex is impaired, and the lower esophageal sphincter tone is reduced. Early EN can cause gastric retention, and vomiting without airway protection may result in the aspiration of gastric contents into the lungs, triggering aspiration pneumonia. Such complications can prolong the infection control period and the duration of mechanical ventilation dependence, ultimately extending ICU length of stay (32).

For septic shock patients requiring high-dose vasopressors, the risk of early EN lies in “insufficient gastrointestinal perfusion leading to intestinal ischemia and feeding intolerance,” while delaying EN beyond 96 h significantly reduces this risk. The core pathophysiological mechanism is that in shock, the body prioritizes blood supply to the heart and brain, leaving the gastrointestinal mucosa in a state of “relative ischemia.” Early EN increases intestinal oxygen demand, exacerbates mucosal ischemic injury, and may even induce non-occlusive intestinal ischemia. Additionally, high stress inhibits gastrointestinal motility, resulting in an EN retention rate of over 40%, which can lead to gastric retention, intestinal distension, and increased risk of enterogenic infections. By delaying EN until beyond 96 h, when the patient’s circulatory status stabilizes and gastrointestinal perfusion improves, the rate of feeding intolerance decreases from 42% in the early group to 18%, and the incidence of enterogenous infections drops from 25 to 10% (33). This significantly avoids prolonged ICU stays due to nutrition-related complications, further supporting the benefit of initiating EN beyond 96 h for septic shock patients in reducing ICU length of stay. The potential advantage of initiating EN beyond 96 h lies in delaying to avoid complications such as aspiration and intestinal ischemia, with nutritional support initiated only after the patient’s physiological condition stabilizes, thereby shortening ICU hospitalization time.

The optimal timing for initiating EN in critically ill patients remains a contentious issue in clinical practice. This controversy has led to conflicting recommendations in clinical guidelines (e.g., some guidelines suggest initiating EN within 24–48 h after achieving hemodynamic stability, while others recommend delaying until after the acute phase). Such inconsistencies directly result in a lack of standardized clinical decision-making, potentially compromising the efficacy and safety of nutritional support in critically ill patients (34). Some researchers argue that early initiation of EN can protect intestinal mucosal barrier function, modulate immune-metabolic homeostasis, and thereby reduce infection risks and improve patient outcomes. Conversely, proponents of delayed EN initiation emphasize that critically ill patients may experience gastrointestinal dysfunction and hemodynamic instability during the acute phase, initiating EN too early could thus increase the risk of complications such as aspiration, diarrhea, or intestinal ischemic injury. This controversy not only reflects differing understandings of the pathophysiological state of critically ill patients but also exacerbates inconsistencies in clinical practice and guideline recommendations. Our network meta-analysis aims to reveal, potential trends and provides new evidence-based perspectives on this debate, while further highlighting the complexity of the conflict.

This study addresses the “early vs. delayed” controversy by systematically comparing the effects of different EN initiation timings via network meta-analysis and revealing potential stratified benefits. However, the credibility of the pooled effect sizes across studies may be limited due to several limitations. First, inadequately described allocation concealment may have introduced selection bias, leading to imbalances in baseline characteristics between groups. Second, the absence of blinding in outcome assessment might have amplified between-group differences due to subjective judgments. These factors collectively reduce the reliability of the combined effect estimates. Additionally, significant heterogeneity in the definitions of “early” and “delayed” across studies—for example, some studies define “early” as <24 h, which differs from the 24–48 h early window proposed in this study—directly increased statistical heterogeneity during evidence synthesis and reduced comparability between studies.

To resolve the “early vs. delayed” controversy and improve the quality of evidence, future research should focus on addressing the following key issues: First, multicenter, large-sample randomized controlled trials (RCTs) should be conducted, providing higher-level evidence through unified study designs and rigorous quality control. Second, collaboration with authoritative institutions is needed to standardize the definitions of EN initiation timings, clarifying criteria for “early” (e.g., within 24–48 h, hemodynamic stability) and “delayed” (e.g., beyond 96 h, high-risk populations) to reduce heterogeneity. Ultimately, these efforts will enable more precise decision-making in nutritional support for critically ill patients.

## 5 Limitations

First, methodological transparency was insufficient across most included trials, particularly regarding allocation concealment (a key determinant of randomization integrity) and blinding of participants, providers, or outcome assessors. These gaps led to an “unclear risk of bias” rating for critical methodological domains, which may introduce imprecision into effect estimates and downgrade evidence certainty per the GRADE framework. Secondly, due to the limited number of included studies and the lack of disease-specific stratification of critically ill patients, this study could only rely on pooled data analysis. This approach prevented a thorough exploration of potential heterogeneity and its impact on the results, thereby limiting the precision of the conclusions and their generalizability to specific clinical subpopulations. Future research should prioritize disease-stratified studies and explore potential treatment effect modifiers to optimize personalized clinical guidance.

## 6 Conclusion

This study provides valuable insights for determining the optimal timing of EN initiation in critically ill patients. EN started within 24–48 h of ICU admission was most effective in reducing mortality. EN initiated after 96 h shortened ICU stays but had low evidence quality and is not recommended. Healthcare providers should integrate patient-specific factors into EN timing decisions, rather than relying solely on evidence-based guidelines.

## Data Availability

The original contributions presented in the study are included in the article/Supplementary material, further inquiries can be directed to the corresponding author.

## References

[1] Elke G Hartl WH Kreymann KG Adolph M Felbinger TW Graf T . Clinical nutrition in critical care medicine - guideline of the German Society for Nutritional Medicine (DGEM). Clin Nutr ESPEN. (2019) 33:220–75. doi: 10.1016/j.clnesp.2019.05.002, 31451265

[2] Vincent JL Marshall JC Namendys-Silva SA François B Martin-Loeches I Lipman J . Assessment of the worldwide burden of critical illness: the intensive care over nations (ICON) audit. Lancet Respir Med. (2014) 2:380–6. doi: 10.1016/S2213-2600(14)70061-X, 24740011

[3] Wu J Lu AD Zhang LP Zuo YX Jia YP. Study of clinical outcome and prognosis in pediatric core binding factor-acute myeloid leukemia. Zhonghua Xue Ye Xue Za Zhi. (2019) 40:52–7. doi: 10.3760/cma.j.issn.0253-2727.2019.01.010 30704229 PMC7351698

[4] McClave SA Martindale RG Vanek VW McCarthy M Roberts P Taylor B . Guidelines for the provision and assessment of nutrition support therapy in the adult critically ill patient: Society of Critical Care Medicine (SCCM) and American Society for Parenteral and Enteral Nutrition (A.S.P.E.N.). JPEN J Parenter Enteral Nutr. (2009) 33:277–316. doi: 10.1177/0148607109335234, 19398613

[5] Cardenas D Bermúdez C Pérez A Diaz G Cortes LY Contreras CP . Nutritional risk is associated with an increase of in-hospital mortality and a reduction of being discharged home: results of the 2009-2015 nutritionDay survey. Clin Nutr ESPEN. (2020) 38:138–45. doi: 10.1016/j.clnesp.2020.05.014, 32690148

[6] Lee MJ Sayers AE Drake TM Singh P Bradburn M Wilson TR . Malnutrition, nutritional interventions and clinical outcomes of patients with acute small bowel obstruction: results from a national, multicentre, prospective audit. BMJ Open. (2019) 9:e029235. doi: 10.1136/bmjopen-2019-029235, 31352419 PMC6661661

[7] Inciong JFB Chaudhary A Hsu HS Joshi R Seo JM Trung LV . Economic burden of hospital malnutrition: a cost-of-illness model. Clin Nutr ESPEN. (2022) 48:342–50. doi: 10.1016/j.clnesp.2022.01.020, 35331511

[8] Gostyńska A Stawny M Dettlaff K Jelińska A. Clinical nutrition of critically ill patients in the context of the latest ESPEN guidelines. Med Kaunas. (2019) 55:770. doi: 10.3390/medicina55120770, 31810303 PMC6955661

[9] Hill A Heyland DK Ortiz Reyes LA Laaf E Wendt S Elke G . Combination of enteral and parenteral nutrition in the acute phase of critical illness: an updated systematic review and meta-analysis. JPEN J Parenter Enteral Nutr. (2022) 46:395–410. doi: 10.1002/jpen.2125, 33899951

[10] Issac A Dhiraaj S Halemani K Thimmappa L Mishra P Kumar B . Efficacy of early enteral nutrition on gastrointestinal surgery outcomes: a systematic review and meta-analysis. Eur J Pediatr Surg. (2023) 33:454–62. doi: 10.1055/s-0043-1760837, 36724826

[11] Wu JY Liu MY Liu TH Kuo CY Hung KC Tsai YW . Clinical efficacy of enteral nutrition feeding modalities in critically ill patients: a systematic review and meta-analysis of randomized controlled trials. Eur J Clin Nutr. (2023) 77:1026–33. doi: 10.1038/s41430-023-01313-8, 37479805

[12] Doig GS Heighes PT Simpson F Sweetman EA Davies AR. Early enteral nutrition, provided within 24 h of injury or intensive care unit admission, significantly reduces mortality in critically ill patients: a meta-analysis of randomised controlled trials. Intensive Care Med. (2009) 35:2018–27. doi: 10.1007/s00134-009-1664-4, 19777207

[13] Ojo O Ojo OO Feng Q Boateng J Wang X Brooke J . The effects of enteral nutrition in critically ill patients with COVID-19: a systematic review and meta-analysis. Nutrients. (2022) 14:1120. doi: 10.3390/nu14051120, 35268095 PMC8912272

[14] Al-Dorzi HM Albarrak A Ferwana M Murad MH Arabi YM. Lower versus higher dose of enteral caloric intake in adult critically ill patients: a systematic review and meta-analysis. Crit Care. (2016) 20:358. doi: 10.1186/s13054-016-1539-3, 27814776 PMC5097427

[15] Lankelma JM van Vught LA Belzer C Schultz MJ van der Poll T de Vos WM . Critically ill patients demonstrate large interpersonal variation in intestinal microbiota dysregulation: a pilot study. Intensive Care Med. (2017) 43:59–68. doi: 10.1007/s00134-016-4613-z, 27837233 PMC5203863

[16] Schuetz P Fehr R Baechli V Geiser M Deiss M Gomes F . Individualised nutritional support in medical inpatients at nutritional risk: a randomised clinical trial. Lancet. (2019) 393:2312–21. doi: 10.1016/S0140-6736(18)32776-4, 31030981

[17] Singer P Blaser AR Berger MM Alhazzani W Calder PC Casaer MP . ESPEN guideline on clinical nutrition in the intensive care unit. Clin Nutr. (2019) 38:48–79. doi: 10.1016/j.clnu.2018.08.037, 30348463

[18] Preiser JC Arabi YM Berger MM Casaer M McClave S Montejo-González JC . A guide to enteral nutrition in intensive care units: 10 expert tips for the daily practice. Crit Care. (2021) 25:424. doi: 10.1186/s13054-021-03847-4, 34906215 PMC8669237

[19] Shim S Yoon BH Shin IS Bae JM. Network meta-analysis: application and practice using Stata. Epidemiol Health. (2017) 39:e2017047. doi: 10.4178/epih.e2017047, 29092392 PMC5733388

[20] Page MJ McKenzie JE Bossuyt PM Boutron I Hoffmann TC Mulrow CD . The PRISMA 2020 statement: an updated guideline for reporting systematic reviews. BMJ. (2021) 372:n71. doi: 10.1136/bmj.n71 33782057 PMC8005924

[21] Cumpston M Li T Page MJ Chandler J Welch VA Higgins JP . Updated guidance for trusted systematic reviews: a new edition of the Cochrane handbook for systematic reviews of interventions. Cochrane Database Syst Rev. (2019) 10:Ed000142. doi: 10.1002/14651858.ED000142 31643080 PMC10284251

[22] Sterne JAC Savović J Page MJ Elbers RG Blencowe NS Boutron I . RoB 2: a revised tool for assessing risk of bias in randomised trials. BMJ. (2019) 366:l4898. doi: 10.1136/bmj.l4898 31462531

[23] Guyatt GH Oxman AD Vist GE Kunz R Falck-Ytter Y Alonso-Coello P . GRADE: an emerging consensus on rating quality of evidence and strength of recommendations. BMJ. (2008) 336:924–6. doi: 10.1136/bmj.39489.470347.AD, 18436948 PMC2335261

[24] Higgins JP Thompson SG Deeks JJ Altman DG. Measuring inconsistency in meta-analyses. BMJ. (2003) 327:557–60. doi: 10.1136/bmj.327.7414.557, 12958120 PMC192859

[25] van Valkenhoef G Dias S Ades AE Welton NJ. Automated generation of node-splitting models for assessment of inconsistency in network meta-analysis. Res Synth Methods. (2016) 7:80–93. doi: 10.1002/jrsm.1167, 26461181 PMC5057346

[26] Rücker G Schwarzer G. Ranking treatments in frequentist network meta-analysis works without resampling methods. BMC Med Res Methodol. (2015) 15:58. doi: 10.1186/s12874-015-0060-8, 26227148 PMC4521472

[27] Nguyen NQ Fraser RJ Bryant LK Burgstad C Chapman MJ Bellon M . The impact of delaying enteral feeding on gastric emptying, plasma cholecystokinin, and peptide YY concentrations in critically ill patients. Crit Care Med. (2008) 36:1469–74. doi: 10.1097/CCM.0b013e31816fc457, 18434906

[28] Mochizuki H Trocki O Dominioni L Brackett KA Joffe SN Alexander JW. Mechanism of prevention of postburn hypermetabolism and catabolism by early enteral feeding. Ann Surg. (1984) 200:297–310. doi: 10.1097/00000658-198409000-00007, 6431918 PMC1250475

[29] Pereira C Murphy K Jeschke M Herndon DN. Post burn muscle wasting and the effects of treatments. Int J Biochem Cell Biol. (2005) 37:1948–61. doi: 10.1016/j.biocel.2005.05.009, 16109499

[30] Zhang J Yu WQ Wei T Zhang C Wen L Chen Q . Effects of short-peptide-based enteral nutrition on the intestinal microcirculation and mucosal barrier in mice with severe acute pancreatitis. Mol Nutr Food Res. (2020) 64:e1901191. doi: 10.1002/mnfr.201901191, 31965752

[31] Nakashima I Horibe M Sanui M Sasaki M Sawano H Goto T . Impact of enteral nutrition within 24 hours versus between 24 and 48 hours in patients with severe acute pancreatitis: a multicenter retrospective study. Pancreas. (2021) 50:371–7. doi: 10.1097/MPA.0000000000001768, 33835968

[32] Jacobs S Chang RW Lee B Bartlett FW. Continuous enteral feeding: a major cause of pneumonia among ventilated intensive care unit patients. JPEN J Parenter Enteral Nutr. (1990) 14:353–6. doi: 10.1177/0148607190014004353, 2119441

[33] Krezalek MA DeFazio J Zaborina O Zaborin A Alverdy JC. The shift of an intestinal "microbiome" to a "pathobiome" governs the course and outcome of sepsis following surgical injury. Shock. (2016) 45:475–82. doi: 10.1097/SHK.0000000000000534, 26863118 PMC4833524

[34] Stolarski AE Young L Weinberg J Kim J Lusczek E Remick DG . Early metabolic support for critically ill trauma patients: a prospective randomized controlled trial. J Trauma Acute Care Surg. (2022) 92:255–65. doi: 10.1097/TA.0000000000003453, 34739002 PMC8792201

[35] Eyer SD Micon LT Konstantinides FN Edlund DA Rooney KA Luxenberg MG . Early enteral feeding does not attenuate metabolic response after blunt trauma. J Trauma. (1993) 34:639–43. discussion 643-634. doi: 10.1097/00005373-199305000-00005 8496997

[36] Watters JM Kirkpatrick SM Norris SB Shamji FM Wells GA. Immediate postoperative enteral feeding results in impaired respiratory mechanics and decreased mobility. Ann Surg. (1997) 226:369–77. discussion 377-380. doi: 10.1097/00000658-199709000-00016 9339943 PMC1191041

[37] Kompan L Kremzar B Gadzijev E Prosek M. Effects of early enteral nutrition on intestinal permeability and the development of multiple organ failure after multiple injury. Intensive Care Med. (1999) 25:157–61. doi: 10.1007/s001340050809, 10193541

[38] Minard G Kudsk KA Melton S Patton JH Tolley EA. Early versus delayed feeding with an immune-enhancing diet in patients with severe head injuries. JPEN J Parenter Enteral Nutr. (2000) 24:145–9. doi: 10.1177/0148607100024003145, 10850938

[39] Ibrahim EH Mehringer L Prentice D Sherman G Schaiff R Fraser V . Early versus late enteral feeding of mechanically ventilated patients: results of a clinical trial. JPEN J Parenter Enteral Nutr. (2002) 26:174–81. doi: 10.1177/0148607102026003174, 12005458

[40] Stein J Schulte-Bockholt A Sabin M Keymling M. A randomized prospective trial of immediate vs. next-day feeding after percutaneous endoscopic gastrostomy in intensive care patients. Intensive Care Med. (2002) 28:1656–60. doi: 10.1007/s00134-002-1473-5, 12415457

[41] Kompan L Vidmar G Spindler-Vesel A Pecar J. Is early enteral nutrition a risk factor for gastric intolerance and pneumonia? Clin Nutr. (2004) 23:527–32. doi: 10.1016/j.clnu.2003.09.013 15297088

[42] Peck MD Kessler M Cairns BA Chang YH Ivanova A Schooler W. Early enteral nutrition does not decrease hypermetabolism associated with burn injury. J Trauma. (2004) 57:1143–8. discussion 1148-1149. doi: 10.1097/01.ta.0000145826.84657.38 15625442

[43] Maude RJ Hoque G Hasan MU Sayeed A Akter S Samad R . Timing of enteral feeding in cerebral malaria in resource-poor settings: a randomized trial. PLoS One. (2011) 6:e27273. doi: 10.1371/journal.pone.0027273, 22110624 PMC3217943

[44] Chourdakis M Kraus MM Tzellos T Sardeli C Peftoulidou M Vassilakos D . Effect of early compared with delayed enteral nutrition on endocrine function in patients with traumatic brain injury: an open-labeled randomized trial. JPEN J Parenter Enteral Nutr. (2012) 36:108–16. doi: 10.1177/0148607110397878, 21965459

[45] Sun JK Mu XW Li WQ Tong ZH Li J Zheng SY. Effects of early enteral nutrition on immune function of severe acute pancreatitis patients. World J Gastroenterol. (2013) 19:917–22. doi: 10.3748/wjg.v19.i6.917, 23431120 PMC3574890

[46] Patel JJ Kozeniecki M Peppard WJ Peppard SR Zellner-Jones S Graf J . Phase 3 pilot randomized controlled trial comparing early trophic enteral nutrition with "no enteral nutrition" in mechanically ventilated patients with septic shock. JPEN J Parenter Enteral Nutr. (2020) 44:866–73. doi: 10.1002/jpen.1706, 31535394

[47] Yu A Xie Y Zhong M Wang F Huang H Nie L . Comparison of the initiation time of enteral nutrition for critically ill patients: at admission vs. 24 to 48 hours after admission. Emerg Med Int. (2021) 2021:3047732. doi: 10.1155/2021/3047732, 34580613 PMC8464429

[48] Wang J Yu K Zeng Y. Early enteral nutrition intervention promotes multiple functional recovery in patients with traumatic intracerebral hemorrhage: a prospective randomized controlled study. Clin Neurol Neurosurg. (2023) 234:108010. doi: 10.1016/j.clineuro.2023.108010, 37857236

