# Supplementary material for: Optimal timing of enteral nutrition initiation in critically ill patients: a network meta-analysis
Source: Front Nutr. 2026 Feb 6;13:1722626. doi: 10.3389/fnut.2026.1722626 (PMC12920217; doi:10.3389/fnut.2026.1722626)

Appendix 1: Search strategies

1. PubMed：296 studies

#1 "Intensive Care Units"[MeSH Terms]

#2 "ICU "[Title/Abstract] OR "close attention unit"[Title/Abstract] OR "critical care unit"[Title/Abstract] OR "general ICU"[Title/Abstract] OR "GICU"[Title/Abstract] OR "GICUs"[Title/Abstract] OR "ICU`s"[Title/Abstract] OR "intensive care department"[Title/Abstract] OR "intensive care units"[Title/Abstract] OR "intensive therapy unit"[Title/Abstract] OR "intensive treatment unit"[Title/Abstract] OR "respiratory care unit"[Title/Abstract] OR "respiratory care units"[Title/Abstract] OR "special care unit"[Title/Abstract] OR "medical ICU"[Title/Abstract] OR "surgical ICU"[Title/Abstract] OR "medical ICUs"[Title/Abstract] OR "surgical ICUs"[Title/Abstract] OR "surgery ICU"[Title/Abstract]

#3 "Enteral Nutrition"[MeSH Terms]

#4 "Tube Feeding"[Title/Abstract] OR "Enteral Feeding"[Title/Abstract] OR "Gastric Feeding Tubes"[Title/Abstract] OR "Gastric Feeding Tube"[Title/Abstract] OR "Force Feeding"[Title/Abstract] OR "Force Feedings"[Title/Abstract] OR "enteric nutrition"[Title/Abstract] OR "intestinal feeding"[Title/Abstract] OR "intragastric feeding"[Title/Abstract] OR "intraintestinal feeding"[Title/Abstract] OR "tube feeding"[Title/Abstract] OR "enteral feeding"[Title/Abstract]

#5 "Randomized controlled trial"[Title/Abstract] OR "Randomized controlled trials"[Title/Abstract] OR "Randomized trials"[Title/Abstract]

#6 (#1 OR #2) AND (#3 OR #4) AND #5

2. Embase：441 studies

#1 'intensive care unit'/exp

#2 'ICU ':ti,ab,kw OR 'close attention unit':ti,ab,kw OR 'critical care unit':ti,ab,kw OR 'general ICU':ti,ab,kw OR 'GICU':ti,ab,kw OR 'GICUs':ti,ab,kw OR 'ICU`s':ti,ab,kw OR 'intensive care department':ti,ab,kw OR 'intensive care units':ti,ab,kw OR 'intensive therapy unit':ti,ab,kw OR 'intensive treatment unit':ti,ab,kw OR 'respiratory care unit':ti,ab,kw OR 'respiratory care units':ti,ab,kw OR 'special care unit':ti,ab,kw OR 'medical ICU':ti,ab,kw OR 'surgical ICU':ti,ab,kw OR 'medical ICUs':ti,ab,kw OR 'surgical ICUs':ti,ab,kw OR 'surgery ICU':ti,ab,kw

#3 'enteric feeding'/exp

#4 'Tube Feeding':ti,ab,kw OR 'Enteral Feeding':ti,ab,kw OR 'Gastric Feeding Tubes':ti,ab,kw OR 'Gastric Feeding Tube':ti,ab,kw OR 'Force Feeding':ti,ab,kw OR 'Force Feedings':ti,ab,kw OR 'enteric nutrition':ti,ab,kw OR 'intestinal feeding':ti,ab,kw OR 'intragastric feeding':ti,ab,kw OR 'intraintestinal feeding':ti,ab,kw OR 'tube feeding':ti,ab,kw OR 'enteral feeding':ti,ab,kw

#5 'randomized controlled trial':ti,ab,kw OR 'randomized controlled trials':ti,ab,kw OR 'randomized trials':ti,ab,kw

#6 (#1 OR #2) AND (#3 OR #4) AND #5

3. Cochrane Library：390 studies

#1 [Intensive Care Units] explode all trees

#2 ("ICU " OR "close attention unit" OR "critical care unit" OR "general ICU" OR "GICU" OR "GICUs" OR "ICU`s" OR "intensive care department" OR "intensive care units" OR "intensive therapy unit" OR "intensive treatment unit" OR "respiratory care unit" OR "respiratory care units" OR "special care unit" OR "medical ICU" OR "surgical ICU" OR "medical ICUs" OR "surgical ICUs" OR "surgery

ICU"):ti,ab,kw

#3 [Enteral Nutrition] explode all trees

#4 ("Tube Feeding" OR "Enteral Feeding" OR "Gastric Feeding Tubes" OR "Gastric Feeding Tube" OR "Force Feeding" OR "Force Feedings" OR "enteric nutrition" OR "intestinal feeding" OR "intragastric feeding" OR "intraintestinal feeding" OR "tube feeding" OR "enteral feeding"):ti,ab,kw

#5 ("Randomized controlled trial" OR "Randomized controlled trials" OR "Randomized trials"):ti,ab,kw

#6 (#1 OR #2) AND (#3 OR #4) AND #5

4.Web of Science：880 studies

#1 "ICU " OR "close attention unit" OR "critical care unit" OR "general ICU" OR "GICU" OR "GICUs" OR "ICU`s" OR "intensive care department" OR "intensive care units" OR "intensive therapy unit" OR "intensive treatment unit" OR "respiratory care unit" OR "respiratory care units" OR "special care unit" OR "medical ICU" OR "surgical ICU" OR "medical ICUs" OR "surgical ICUs" OR "surgery ICU"

#2 "Enteral Nutrition " OR "enteric feeding" OR "Tube Feeding" OR "Enteral Feeding" OR "Gastric Feeding Tubes" OR "Gastric Feeding Tube" OR "Force Feeding" OR "Force Feedings" OR "enteric nutrition" OR "intestinal feeding" OR "intragastric feeding" OR "intraintestinal feeding" OR "tube feeding" OR "enteral feeding"

#3 "Randomized controlled trial" OR "Randomized controlled trials" OR "Randomized trials"

#4 #1 AND #2 AND #3

Table2. Grading of Recommendation, Assessment, Development, and Evaluation assessment

Outcome	Studies，n	Quality assessment					Patients(T[a]/C[b])		Effect,mean Difference (95%CI)	Overall Certainty Of evidence
		Risk of bias	Inconsistency	Indirectness	Imprecision	Publication bias	events/total	events/total		
mortality rate	11	Serious[c]	Not serious	Not serious	Not serious	None	58/357 (16.2%)	73/351 (20.8%)	0.74(0.5 to 1.1)	Moderate
Length of stay	12	Not serious	Not serious	Not serious	Serious[d]	None	323/317		1.29(0.76 to 1.82)	Moderate

a:Treatment Group

b:Control Group

c:Allocation concealment and blinding were subject to substantial bias

d:The sample size was small, leading to wide confidence intervals.

Appendix 2: Inconsistency test

Table A1 Inconsistency test in mixed intervention comparison

**Outcomes**	**Loops**	**IF**	**Z**	**P**	**95%CI**	**Loop_Heterog _tau^2**
mortality rate	B-C-E	2.810	1.662	0.096	(0.00,6.12)	0.000
chi^2(3)= 3.22	A-B-E	1.088	0.855	0.392	(0.00,3.58)	0.000
P= 0.3584	A-C-E	0.921	0.774	0.439	(0.00,3.25)	0.028
	A-B-C	0.518	0.335	0.737	(0.00,3.54)	0.855
Length of ICU stay	A-B-E	8.925	2.130	0.033	(0.71,17.14)	5.828
chi^2(3)= 7.83	B-C-E	7.900	1.820	0.069	(0.00,16.41)	0.000
P= 0.0496	A-B-C	5.740	2.306	0.021	(0.86,10.62)	0.000
	A-C-E	4.592	0.933	0.351	(0.00,14.24)	6.337

A,B,C,D,E represent 5 time points for enteral nutrition in critically ill patient

IF: Inconsistency Factor

*Loop_Heterog_tau2: Variance of true effect size distribution of heterogeneity between studies in loop.

Appendix 3: Network plots

I

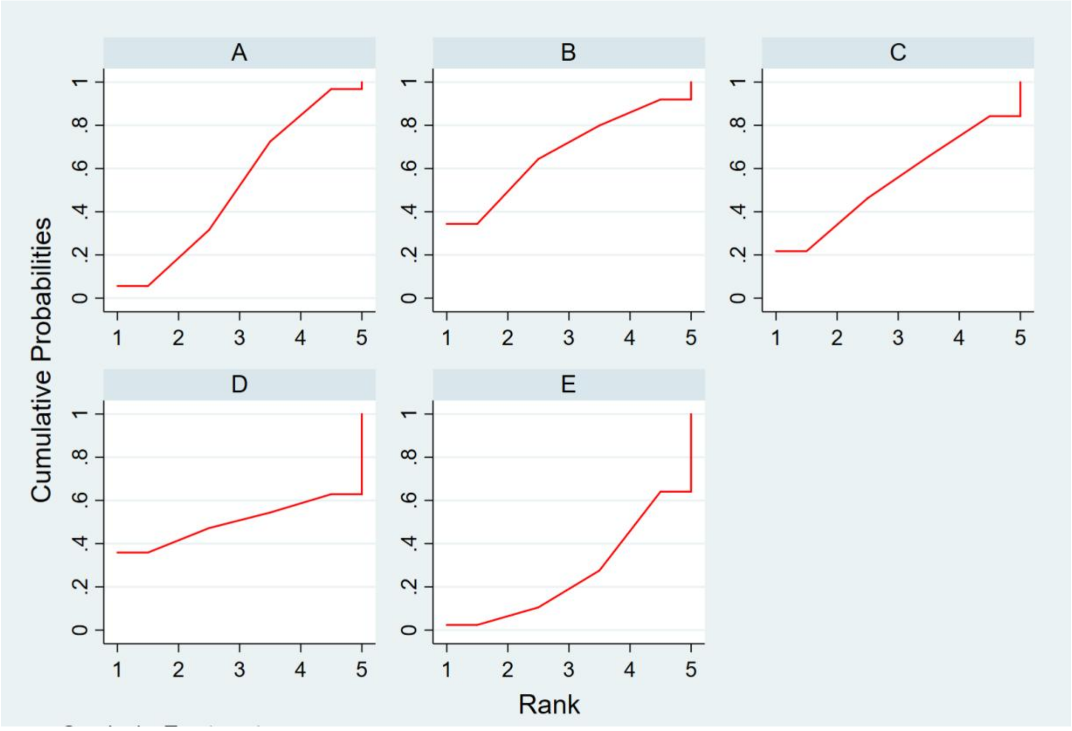

II

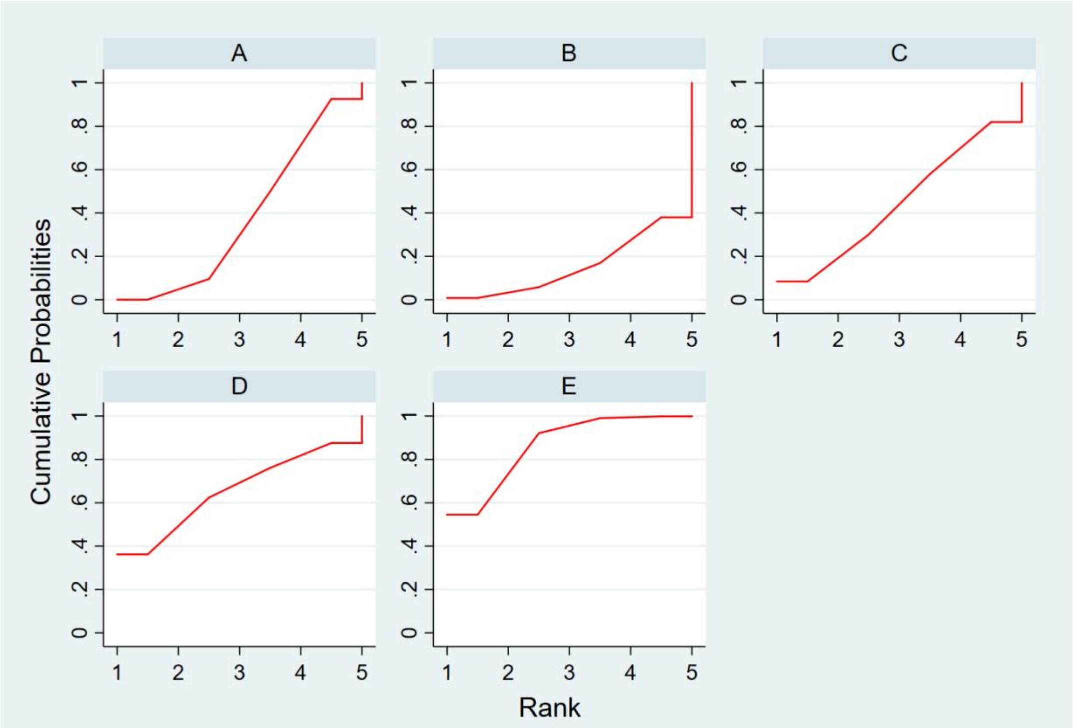

Supplement: Supplementary file 1 [file Data_Sheet_1.pdf]
